# Prognostic value of combining 24-hour ASPECTS and hemoglobin to red cell distribution width ratio to the THRIVE score in predicting in-hospital mortality among ischemic stroke patients treated with intravenous thrombolysis

**DOI:** 10.1371/journal.pone.0304765

**Published:** 2024-06-25

**Authors:** Sarawut Krongsut, Surachet Srikaew, Niyada Anusasnee

**Affiliations:** 1 Division of Neurology, Department of Internal Medicine, Saraburi Hospital, Saraburi, Thailand; 2 Division of Neurosurgery, Department of Surgery, Faculty of Medicine, Srinakharinwirot University, Ongkharak Campus, Nakhon Nayok, Thailand; 3 Division of radiology, Saraburi Hospital, Saraburi, Thailand; UCSF: University of California San Francisco, UNITED STATES

## Abstract

**Background:**

Acute ischemic stroke (AIS) is a significant global health issue, directly impacting mortality and disability. The Totaled Health Risks in Vascular Events (THRIVE) score is appreciated for its simplicity and ease of use to predict stroke clinical outcomes; however, it lacks laboratory and neuroimaging data, which limits its ability to predict outcomes precisely. Our study evaluates the impact of integrating the 24-hour Alberta Stroke Program Early CT Score (ASPECTS) and hemoglobin-to-red cell distribution width (HB/RDW) ratio into the THRIVE score using the multivariable fractional polynomial (MFP) method (combined THRIVE-MFP model) compared to the THRIVE-c model. We aim to assess their added value in predicting in-hospital mortality (IHM) prognosis.

**Materials and methods:**

A retrospective study from January 2015 to July 2022 examined consecutive AIS patients receiving intravenous thrombolysis. Data on THRIVE scores, 24-hour ASPECTS, and HB/RDW levels were collected upon admission. The model was constructed using logistic regression and the MFP method. The prognostic value was determined using the area under the receiver operating characteristic curve (AuROC). Ischemic cerebral lesions within the middle cerebral artery territory were evaluated with non-contrast computed tomography (NCCT) after completing 24 hours of intravenous thrombolysis (24-hour ASPECTS).

**Results:**

Among a cohort of 345 patients diagnosed with AIS who received intravenous thrombolysis, 65 individuals (18.8%) experienced IHM. The combined THRIVE-MFP model was significantly superior to the THRIVE-c model in predicting IHM (AuROC 0.980 vs. 0.876, *p*<0.001), 3-month mortality (AuROC 0.947 vs. 0.892, *p*<0.001), and 3-month poor functional outcome (AuROC 0.910 vs. 0.853, *p*<0.001).

**Conclusion:**

The combined THRIVE-MFP model showed excellent predictive performance, enhancing physicians’ ability to stratify patient selection for intensive neurological monitoring and guiding treatment decisions. Incorporating 24-hour ASPECTS on NCCT and HB/RDW proved valuable in mortality prediction, particularly for hospitals with limited access to advanced neuroimaging resources.

## 1. Introduction

Ischemic stroke is a significant cause of global mortality, ranking second, and it is a leading cause of disability, despite advancements in treatment [1]. In recent decades, the prevalence of ischemic stroke has shifted from developed countries to developing countries [2]. Thailand’s disability and mortality rates vary from 10% to 50%. The Ministry of Public Health implements innovative technological policies to reduce associated costs and improve outcomes [3]. Intravenous recombinant tissue plasminogen activator (rt-PA) is the main treatment for acute ischemic stroke (AIS) within 3–4.5 hours of symptom onset. While it improves outcomes, it doesn’t lower mortality rates [4]. Early detection, vigilant monitoring, and appropriate risk stratification measures are essential for optimizing stroke unit management and improving prognosis in AIS patients.

Recently, numerous mortality prediction models for AIS have emerged [5–7], but the accuracy of these models in routine clinical practice has not been thoroughly investigated, so their applicability remains limited. Practical clinical risk tools that accurately predict mortality in ischemic stroke are needed. The Totaled Health Risks in Vascular Events (THRIVE) score is an easily applicable stroke outcome prediction tool that considers clinical predictors available at the time of stroke presentation, including age, National Institutes of Health Stroke Scale (NIHSS), and Chronic Disease Scale (CDS) factors including hypertension (HTN), diabetes mellitus (DM), or atrial fibrillation (AF). It was developed using data from the MERCI and Multi-MERCI trials [8]. Continuous variables, like age, are categorized into dichotomized or trichotomized groups to simplify score calculation and facilitate the use of predictive scores without relying on computer-based tools. However, these simplifications may compromise the accuracy of outcome prediction. In a previous study, Alexander C. Flint et al. showed that the THRIVE-c calculation, using continuous variables and a logistic equation, outperformed the traditional THRIVE score in predicting poor outcomes (modified Rankin Scale [mRS] 3–6 at 90 days) with a higher area under the curve (AuROC) (AuROC = 0.785, 95% CI: 0.777–0.793 vs. AuROC = 0.746, 95% CI: 0.737–0.755) and a significant *p*-value of less than 0.001 [9].

The Alberta Stroke Program Early CT Score (ASPECTS) is a reliable and clinically applicable system for assessing brain computed tomography (CT) images. It accurately identifies the location of early-stage AIS, particularly in cases involving middle cerebral artery occlusion [10]. Previous research has shown that the 24-hour ASPECTS, assessed through non-contrast computed tomography (NCCT) at 24 hours after administering rt-PA, demonstrates superior predictive ability compared to the pre-thrombolysis ASPECTS in predicting the functional stroke outcome at 3 months [11, 12]. Although Diffusion-weighted imaging ASPECTS demonstrates superior sensitivity in the early detection of ischemic changes after stroke onset compared to NCCT-ASPECTS [13], the utilization of CT perfusion and magnetic resonance imaging for prompt diagnosis and management of AIS patients remains limited in routine clinical practice in Thailand due to resource and cost constraints.

Low blood oxygen saturation is linked to increased red cell distribution width (RDW), potentially impacting ischemic stroke development by reducing brain oxygen delivery. RDW also exhibits a significant statistical association with AIS severity, indicating its potential as a predictor for AIS prognosis [14, 15]. Currently, the hemoglobin to red cell distribution width (HB/RDW) ratio has emerged as a significant prognostic tool for predicting mortality in heart failure, cardiovascular hospitalizations [16], survival outcomes in cancer patients [17], and risk of death in patients with AF and AIS [18]. Therefore, the 24-hour ASPECTS and HB/RDW ratio may serve as easily applicable, non-invasive, and cost-effective predictors. They encompass the diverse clinical characteristics of patients and should be considered to enhance the efficacy of clinical prediction for in-hospital mortality (IHM).

With widespread computer and internet access, simplifying tools by setting cut-off values for continuous predictors is unnecessary. Multivariable fractional polynomial (MFP) algorithms are used for continuous predictors to identify optimal polynomial transformations, ensuring a good fit in the binary logistic model and generating regression coefficients. These coefficients create a logistic equation for individualized prediction, facilitating outcome probability analysis. Utilizing computer or mobile applications enables the calculation of probabilities based on equations derived from the implementation of the MFP procedure, which may provide greater accuracy than using continuous variables and the logistic equation. However, limited data exists on the clinical significance of 24-hour ASPECTS and HB/RDW ratio regarding IHM in AIS patients treated with rt-PA.

This study aimed to evaluate the added prognostic value of incorporating 24-hour ASPECTS and HB/RDW into the THRIVE score through the MFP procedure (combined THRIVE-MFP), as compared to the THRIVE-c model. Additionally, it sought to develop a novel clinical prediction model by combining the THRIVE score with 24-hour ASPECTS and HB/RDW to predict IHM in AIS patients treated with rt-PA.

## 2. Methods

### 2.1. Study population

We employed a retrospective cohort design for our study, gathering data from electronic medical records of 345 patients diagnosed with AIS who received intravenous thrombolysis within the 3–4.5-hour window from symptom onset or last known normal. The research was conducted at Saraburi Hospital between January 2015 and July 2022. The selection guidelines for individuals suitable for rt-PA were applied in accordance with the 2019 early management guidelines for AIS [19]. The inclusion criteria were: (1) age ≥ 18 years; (2) diagnosis of acute anterior circulation ischemic stroke (AACIS); and (3) patients treated with rt-PA only. Exclusion criteria included: (1) diagnosis of transient ischemic attack or minor stroke; (2) being pregnant or lactating; (3) posterior circulation ischemic strokes; (4) poor-quality NCCT scans; (5) referred patients with untracked treatment data; (6) incomplete clinical information for THRIVE score; (7) patients deceased within 24 hours after the initiation of rt-PA. AIS patients receiving EVT were not included in this study due to the unavailability of a neuro-interventionalist at our center. Patients meeting the criteria for EVT were not referred to another center due to reimbursement constraints in Thailand’s public health policy during the study period, thereby hindering eligible EVT candidates from accessing this treatment elsewhere. All patients were admitted to the hospital, irrespective of the presence of large vessel occlusion. The information was completely anonymized prior to our access, and the ethics committee waived the requirement for informed consent. We don’t gather any patient-identifying details, such as hospital numbers, admission numbers, identity card numbers, or birthdates. The study protocol (Approval ID. EC034/2566) received approval from the Institutional Review Board and Ethics Committee of Saraburi Hospital on July 24, 2023. The data was retrieved for research purposes on July 30, 2023. The provided URL affords access to research article protocols accessible on the protocols.io platform: https://dx.doi.org/10.17504/protocols.io.dm6gp3o5pvzp/v1.

### 2.2. Data collection

Patient demographics (gender, age), vascular risk factors (DM, HTN, AF), HB/RDW, and 24-hour ASPECTS were obtained from medical records. Upon hospital admission, hemoglobin (Hb) levels and RDW were also assessed. The Sysmex XN-3000 automated analyzer was used to measure complete blood count parameters. Upon admission, NIHSS was employed to evaluate the severity of strokes. Certified neurologists and neuroradiologists evaluated the 24-hour ASPECTS, and discrepancies were resolved through consultation and consensus to ensure accurate results.

### 2.3. THRIVE-c and combined THRIVE-MFP calculation

The THRIVE-c calculation was a multivariable logistic regression (MVLR) model constructed by entering continuous inputs of age and NIHSS and combining them with the dichotomous inputs for HTN, DM, and AF for estimated outcome probability for individual patients.

The combined THRIVE-MFP calculation utilized the MVLR with MFP model, which employed MFP algorithms to optimize the transformation of continuous predictors (age, NIHSS, 24-hour ASPECTS, and HB/RDW). This model also incorporated dichotomous inputs for HTN, DM, and AF, resulting in the most accurate calculation of the predicted outcome probability.

### 2.4. Outcomes assessment

The primary endpoint of this study was IHM, which included death from all causes following intravenous thrombolysis. Patient survival information was obtained from discharge summaries, and admissions were classified as either non-survivors or survivors. 3-month mortality referred to death within 90 days regardless of causes, and 3-month poor functional outcome was defined as mRS > 2 at 90 days after a stroke. All enrolled individuals underwent follow-up via telephone interviews conducted by healthcare professionals specialized in stroke care, including nurses with stroke training and/or physical therapy staff, at the 90-day interval following the stroke event.

### 2.5. Statistical analysis

The statistical software Stata version 16 (StataCorp, College Station, TX, USA) was used for the analyses. Non-normally distributed data were summarized using medians and interquartile ranges (IQR). Normally distributed data were presented as mean ± standard deviation. Categorical variables were presented using frequency distributions and compared using Fisher’s exact test or chi-square analysis. Continuous variables were compared using either the Mann-Whitney U test or the student’s t-test.

#### 2.5.1. Analysis of added value in prediction

We evaluated the additional prognostic value of adding 24-hour ASPECTS and HB/RDW to the THRIVE score for predicting the mortality outcome in AACIS patients treated with rt-PA. Two distinct logistic regression models were developed for the patient cohort. Model 1, known as the THRIVE-c calculation, involved estimating model-predicted probabilities using a logistic equation. The MVLR model was constructed by including variables such as continuous age, continuous NIHSS, and dummy variables for CDS. Model 2, known as the combined THRIVE-MFP calculation, included 24-hour ASPECTS and HB/RDW. MFP algorithms determined the optimal fractional polynomial transformation of each continuous variable for best fit in the binary logistic model. Age, NIHSS, 24-hour ASPECTS, and HB/RDW were included as first-degree fractional polynomial terms.

#### 2.5.2. Development and performance of prediction models

If the combined THRIVE-MFP model demonstrated superior discriminatory ability compared to the THRIVE-c model, it could be advanced as a prognostic prediction model for further evaluation of its performance. Logistic regression equations were used to construct each prediction model. The linear predictor (z) underwent an inverse logit transformation, specifically using the formula e^z^/(1 + e^z^), where e represented the base value of natural logarithms. This transformation approximated the estimated probability of IHM. The performance in prediction was assessed through the analysis of the receiver operating characteristic (ROC) curve. The calibration plot assessed the agreement between the predicted probability of the model and the observed proportions of IHM occurrence. Hosmer-Lemeshow goodness-of-fit statistical analysis was employed to evaluate the model calibration. The prognostic model underwent internal validation through bootstrapping resampling with 500 replicates. We conducted calculations to determine and reported apparent AuROC, test AuROC, large-scale calibration, calibration slope, and shrinkage factor.

A decision curve analysis (DCA) was performed to evaluate the clinical utility and benefits of the THRIVE-c and combined THRIVE-MFP model. The analysis employed the net benefit (NB) approach to identify models with the highest aggregate benefits for patient application and healthcare treatment decisions. The NB, calculated by subtracting false positives from true positives at varying probabilities, guided the determination of optimal models. It represents the minimum probability of outcome occurrence at which further intervention would be necessary. Decision curves were plotted to illustrate the fluctuation in NB for the prediction models across the range of threshold probabilities for high-risk patients [20]. The threshold probability represents the minimum probability of IHM that physicians and nurses must closely monitor for the occurrence of severe stroke complications. The predictive model’s strength lies in its ability to accurately identify individuals at risk of IHM and those who are not.

#### 2.5.3. Comparative analysis of prognostic accuracy between models

We analyzed the predictive values of the THRIVE-c model and the combined THRIVE-MFP model at different levels of predicted risk probabilities. Sensitivity, specificity, positive predictive values (PPV), negative predictive values (NPV), and positive likelihood ratios (LR+) were determined and compared.

## 3. Results

### 3.1. Analysis of baseline characteristics in the study population

Among a cohort of 345 patients diagnosed with AACIS who received treatment with rt-PA, 65 individuals (18.8%) experienced IHM, while 280 patients (81.2%) survived. The median NIHSS score at admission was 11, and the median length of hospital stay was 5 days (IQR 3, 9 days). Table 1 presents a comparison between IHM and survivors of clinical characteristics assessed by the THRIVE score. IHM patients were significantly older (67.5±15.4 vs. 60.45±14.9, *p* = 0.001), had higher prevalence of AF (55.4% vs. 23.9%, *p*<0.001), and had higher NIHSS scores at admission (18.6±4.1 vs. 11.1±5.0, *p*<0.001). However, the proportions of patients with DM and HTN were not significantly different (24.6% vs. 27.5%, *p* = 0.637; and 75.4% vs. 69.3%, *p* = 0.637, respectively). Additionally, 24-hour ASPECTS and HB/RDW were significantly lower (2, IQR: 1–4 vs. 9, IQR: 7–10, *p*<0.001; and 0.76, IQR: 0.64–0.93 vs. 0.89, IQR: 0.79–1.01, *p*<0.001, respectively).

**Table 1:** Patient clinical characteristics.

Characteristics	All patients (n = 345)		IHM (n = 65)		Survived (n = 280)		*p*-value
	n (%)		n (%)		n (%)		
Age (years)—mean (± SD)	61.78 ± 15.23		67.51 ± 15.40		60.45 ± 14.90		0.001
Gender							
Female	162	(47.0)	32	(49.2)	130	(46.4)	0.683
Male	183	(53.0)	33	(50.8)	150	(53.6)	
**Vascular risk factor and comorbidities**							
Atrial fibrillation	103	(29.9)	36	(55.4)	67	(23.93)	<0.001
Diabetes mellitus	93	(27)	16	(24.6)	77	(27.5)	0.637
Hypertension	243	(70.4)	49	(75.4)	194	(69.3)	0.332
**Time to rt-PA (hours)**							
< 3 hours	233	(67.5)	39	(60.0)	194	(69.3)	0.150
3–4.5 hours	112	(32.5)	26	(40.0)	86	(30.7)	
**NIHSS at admission**	12.51 ± 5.66		18.62 ± 4.05		11.09 ± 5.01		<0.001
Hospital stays (days)	5	(3–9)	8	(3–22)	5	(4–8)	0.016
**Laboratory**							
Hb (g/dL)—mean (± SD)	12.55 ± 2.13		11.88 ± 2.30		12.71 ± 2.07		0.009
RDW (%)—mean (± SD)	14.75 ± 2.21		15.89 ± 2.93		14.49 ± 1.91		<0.001
HB/RDW—median (IQR)	0.87		0.76		0.89		<0.001
	(0.76–1.00)		(0.64–0.93)		(0.79–1.01)		
**Workflow time**							
Onset to door (min)—median (IQR)	90		90		90		0.254
	(60–120)		(60–130)		(60–120)		
Onset to treatment time (min)—median (IQR)	42		39		43		0.708
	(28–63.5)		(29–63)		(28–63.5)		
**ASPECTS**							
24-hour ASPECTS	8		2		9		<0.001
	(5–9)		(1–4)		(7–10)		

**Abbreviations:** ASPECTS, Alberta Stroke Program Early CT Score; Hb, hemoglobin; HB/RDW, hemoglobin to red cell distribution width ratio; IHM, in-hospital mortality; IQR, interquartile range; NIHSS, National Institute of Health Stroke Scale; RDW, red cell distribution width; rt-PA, recombinant tissue plasminogen activator; SD, standard deviation.

### 3.2. Exploring the added value of 24-hour ASPECTS and HB/RDW

**Fig 1:**
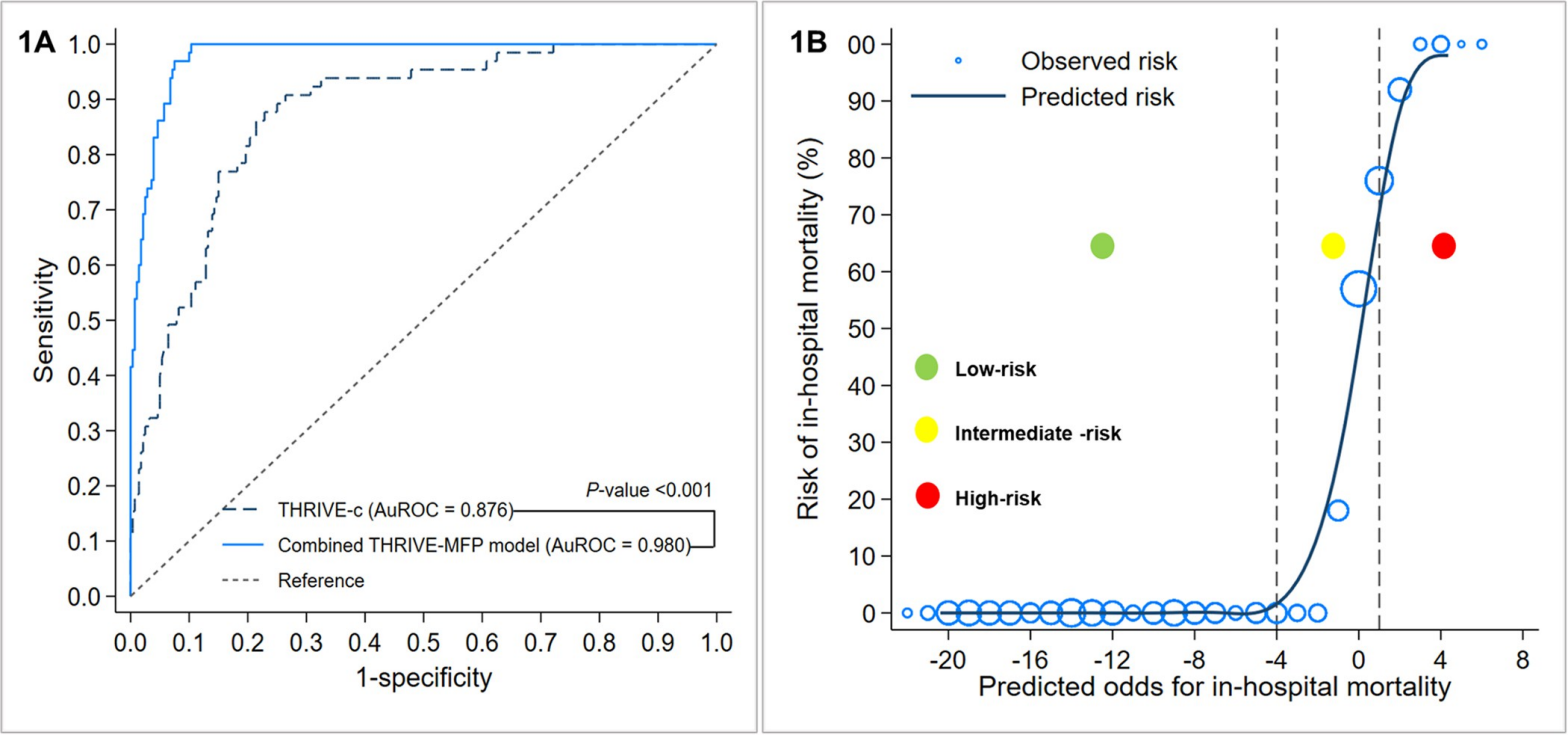
(a) Comparison of ROC curves and the evaluation of performance between the combined THRIVE-MFP model and THRIVE-c model for predicting IHM; (b) Model calibration plot illustrating the agreement between predicted odds and observed proportion of thrombolyzed AACIS patients.

The impact of the prognostic value of 24-hour ASPECTS and HB/RDW on the THRIVE score is presented in Tables 2 and 3. The THRIVE-c and combined THRIVE-MFP model demonstrated predictive performance in distinguishing between IHM and survivors, with AuROC values of 0.876 (95% CI: 0.832, 0.919) and 0.980 (95% CI: 0.969, 0.991), respectively (Fig 1A). The model displayed a notably accurate calibration, as evidenced by the calibration plot (Fig 1B). This plot effectively compared the model’s predicted risk with the actual observed proportion of IHM in each cell of the graph. Table 3 displays the comparison of metrics for model discrimination and reclassification between the THRIVE-c and combined THRIVE-MFP models, indicating that the combined THRIVE-MFP model outperformed the THRIVE-c model (*p*<0.001).

**Table 2:** Evaluation of incorporating 24-hour ASPECTS and HB/RDW with the THRIVE score using fractional polynomial transformation for predicting IHM.

Model / Predictors Included	Optimal FP transformations	β	(95%CI)	*p*-value	AuROC (95%CI)	
**Model A** ^†^ **: THRIVE-c**					0.876	(0.832–0.919)
Age	—	0.011	(-0.012, 0.035)	0.340		
NIHSS	—	0.282	(0.207, 0.358)	<0.001		
Diabetes mellitus	—	-0.076	(-0.869, 0.718)	0.852		
Hypertension	—	0.311	(-0.481, 1.104)	0.441		
Atrial fibrillation	—	0.873	(0.193, 1.553)	0.012		
Intercept (constant)		-6.991	(-8.927, -5.055)			
**Model B** ^‡^ **: Combined THRIVE-MFP model**					0.980	(0.969–0.991)
Age	Age—61.777	0.031	(-0.005, 0.068)	0.091		
NIHSS	NIHSS—12.507	0.109	(-0.021, 0.240)	0.101		
24-hour ASPECTS	([ASPECTS+1]/10)3–0.506	-14.648	(-20.345, -8.951)	<0.001		
HB/RDW	HB/RDW—0.871	-7.059	(-10.446, -3.673)	<0.001		
Diabetes mellitus	Original binary form	-0.744	(-1.938, 0.450)	0.222		
Hypertension	Original binary form	0.226	(-0.962, 1.414)	0.709		
Atrial fibrillation	Original binary form	0.388	(-0.693, 1.468)	0.482		
Intercept (constant)		-6.218	(-8.689, -3.747)			

**Abbreviations:** AuROC, area under the receiver operating characteristic curve; ASPECTS, Alberta stroke program early CT score; FP, fractional polynomial; HB/RDW, hemoglobin to red cell distribution width ratio; IHM, in-hospital mortality; MFP, multivariable fractional polynomial; NIHSS, National Institute of Health Stroke Scale; THRIVE, Totaled Health Risks in Vascular Events.

^†^Model A includes THRIVE-c model

^‡^Model B includes THRIVE score with 24-hour ASPECTS and HB/RDW using MFP algorithm (combined THRIVE- MFP model).

**Table 3:** Improvement in model performance by combined THRIVE-MFP model.

Metrics	Value		*p*-value
AuROC			
Model A: THRIVE-c model	0.876	(0.832–0.919)	
Model B: Combined THRIVE-MFP model	0.980	(0.969–0.991)	
Model A^†^ vs. Model B^‡^			
Δ AuROC	0.104	(0.064–0.145)	<0.001
IDI			
Absolute	0.372	(0.296–0.449)	<0.001
Relative	2.08		
NRI			
Overall	0.368	(0.203–0.533)	<0.001
Events correctly reclassified	35.4%		
Nonevents correctly reclassified	1.4%		

**Abbreviations:** AuROC, area under the receiver operating characteristic curve; ASPECTS, Alberta stroke program early CT score; combined THRIVE-MFP model, combined Totaled Health Risks in Vascular Events ‐multivariable fractional polynomial model; IDI, integrated discrimination improvement; NRI, Net reclassification index; THRIVE-c model, Totaled Health Risks in Vascular Events ‐calculation model.

^†^Model A includes THRIVE-c model

^‡^Model B includes THRIVE score with 24-hour ASPECTS and HB/RDW using MFP algorithm (combined THRIVE-MFP model).

### 3.3. Model prognostic accuracy

The combined THRIVE-MFP model demonstrated significantly superior discriminative ability compared to THRIVE-c. This model incorporated three sets of predictors: (1) the THRIVE score, (2) 24-hour ASPECTS, and (3) HB/RDW. The probability of IHM can be estimated using the combined THRIVE-MFP model through the following equation: e^z^/(1 + e^z^), where z = -6.218 + 0.031 (Age—61.777) + 0.109 (NIHSS—12.507) + -14.648 (([24-hour ASPECTS+1]/10)^3^–0.506) + -7.059 (HB/RDW—0.871) + -0.744 (DM: No = 0 or Yes = 1) + 0.226 (HTN: No = 0 or Yes = 1) + 0.388 (AF: No = 0 or Yes = 1) (Table 2).

**Fig 2:**
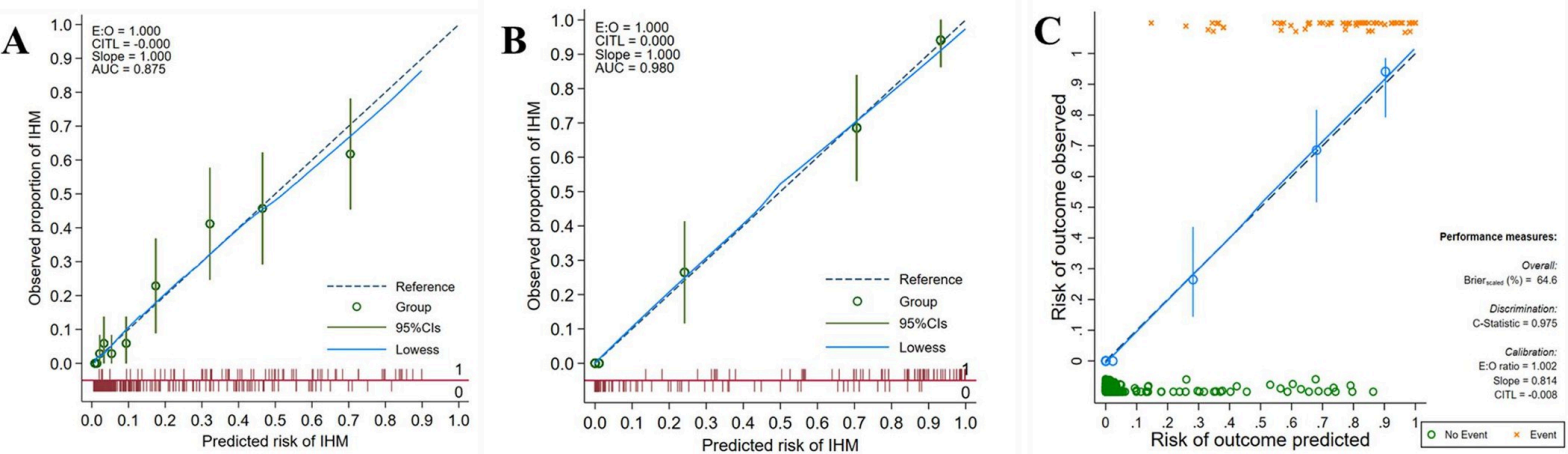
Calibration plot comparing the predicted risk of IHM from (a) THRIVE-c model, (b) combined THRIVE-MFP model, and (c) Internal validation of combined THRIVE-MFP model was performed using 500 bootstrap resamples. Note: The 45-degree straight line provides an ideal agreement between the observed and predicted probability. The vertical bars reflect the 95% CI of the actual probability.

Comparatively, the apparent AuROC of the THRIVE-c model was 0.876. The optimism corrected was 0.864 (95% CI: 0.823, 0.907). The calibration plot (Fig 2A–2C) serves as a visual representation to demonstrate the agreement between the predicted IHM probability from both prediction models and the observed IHM proportion. The combined THRIVE-MFP model showed excellent internal calibration according to the calibration plot (Fig 2B and 2C). The Hosmer-Lemeshow goodness-of-fit statistics were non-significant (no statistical evidence of lack-of-fit) (*p* = 1.00), demonstrating an excellent model fit to the observed data. After internal validation of the combined THRIVE-MFP model using 500 bootstrap resamples, the results revealed an apparent AuROC of 0.980 (95% CI: 0.969, 0.991) and the optimism corrected was 0.974 (95% CI: 0.963, 0.987). The calibration in the large was -0.001 (95% CI: -0.590, 0.554), with a calibration slope of 0.803 (95% CI: 0.503, 1.100). The shrinkage factor was 0.803.

**Fig 3:**
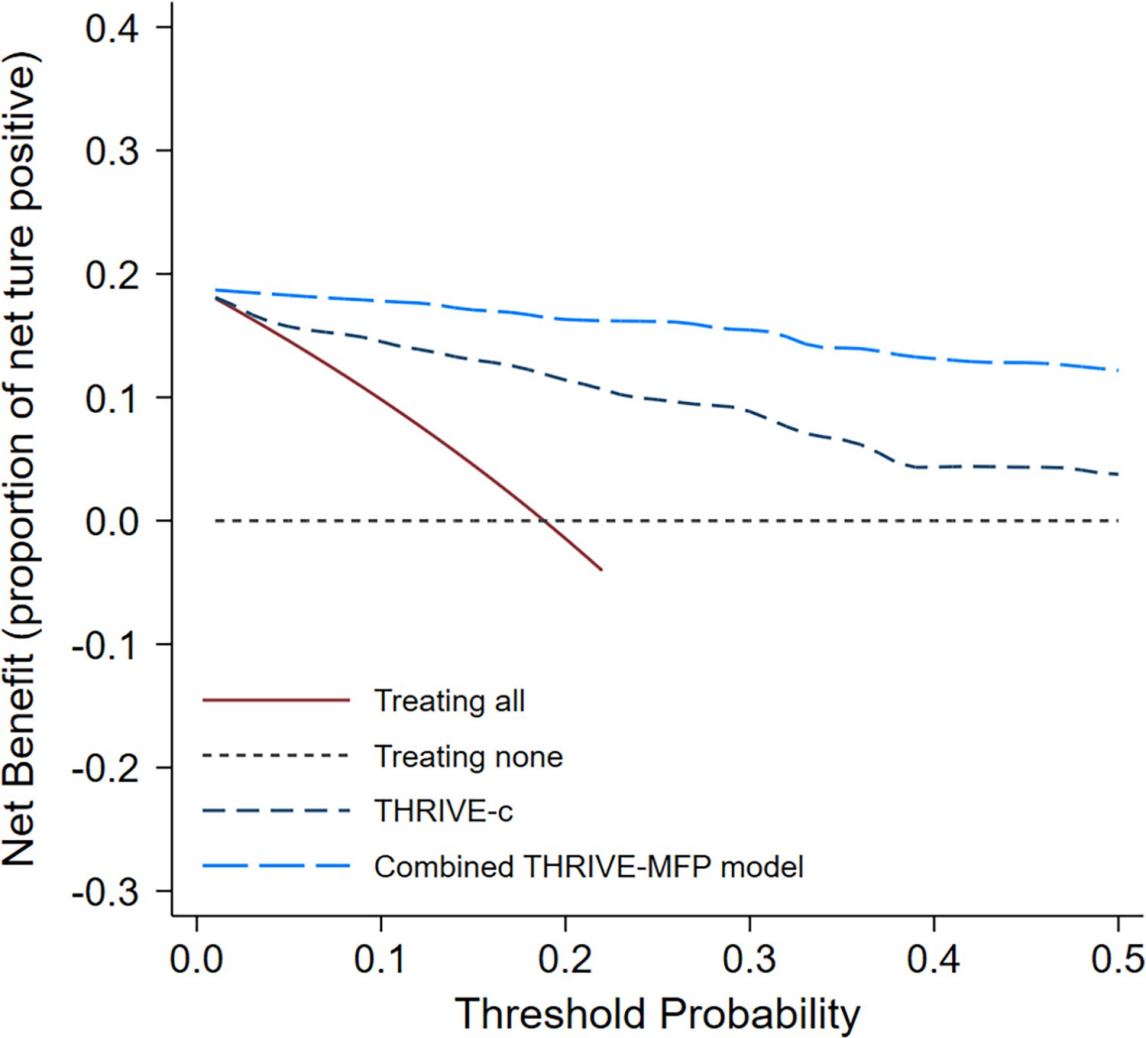
Decision curve analysis illustrating the net benefit of the combined THRIVE-MFP compared to the THRIVE-c model for predicting IHM.

The decision-curve analysis demonstrated the clinical utility of the prognostic models (Fig 3). Both the THRIVE-c and combined THRIVE-MFP models exhibited NB compared to the default strategies, which involved either intensive monitoring of all AIS patients or no monitoring of any patient, across the entire range of threshold probabilities for monitoring high-risk patients. Furthermore, the combined THRIVE-MFP model displayed a higher NB than the THRIVE-c model beyond the threshold probability.

**Fig 4:**
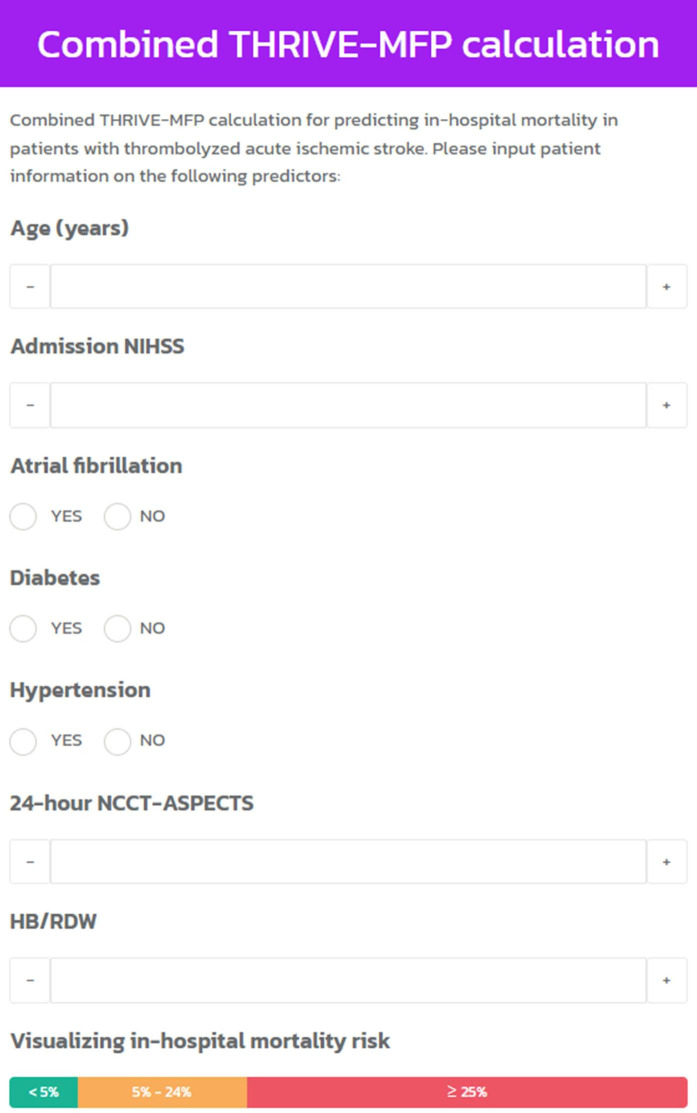
The web application provides free access to the combined THRIVE-MFP model for stratifying the risk of in-hospital mortality in thrombolyzed AACIS patients.

In the context of comparative validation of prognostic performance, our findings revealed that the combined THRIVE-MFP model outperforms the THRIVE-c model in terms of sensitivity, specificity, PPV, NPV, LR+, and accuracy at different predicted risk thresholds. The prognostic index of both models varies based on the predicted risk for IHM. S1 Table presents the sensitivity, specificity, PPV, NPV, and LR+ for each predicted risk threshold. In order to apply the model in clinical settings, we divided the predicted probabilities into three clinical risk categories: low-risk (<5.0%), intermediate-risk (5.0–25.0%), and high-risk (≥ 25%). S2 Table displays the PPV and LR+ for each risk category. The enhanced predictive model is now accessible through this web link: https://www.sbh.go.th/thrive-mfp/ (retrieved on September 25, 2023), facilitating IHM prediction using the combined THRIVE-MFP calculation web application (Fig 4).

Furthermore, both the THRIVE-c and combined THRIVE-MFP models demonstrated predictive efficacy for 3-month mortality and 3-month poor functional outcome. This is supported by the AuROC values of each model and the estimated probability derived from the combined THRIVE-MFP model using the equation e^z^/(1 + e^z^) (S3 and S4 Tables). Comparative analysis of ROC curves (S1 and S2 Figs), calibration plots comparing predicted risk (S3 and S4 Figs), and decision curve analysis illustrating the net benefit (S5 Fig) are provided. The combined THRIVE-MFP model was significantly superior to the THRIVE-c model in predicting 3-month mortality (AuROC 0.947 vs. 0.892, *p*<0.001) and 3-month poor functional outcome (AuROC 0.910 vs. 0.853, *p*<0.001). The Hosmer-Lemeshow goodness-of-fit statistics were non-significant (*p* = 0.703 and 0.209, respectively) (S3 and S4 Tables).

## 4. Discussion

Based on the presented study, the incorporation of 24-hour ASPECTS and HB/RDW with the THRIVE score has demonstrated a significant improvement in prognostic performance for predicting IHM among AACIS patients receiving thrombolysis. The clinical prediction model, known as the combined THRIVE-MFP model using MVLR and MFP algorithm, combines predictors from the THRIVE score, 24-hour ASPECTS, and HB/RDW. To our knowledge, this study is the first to integrate the 24-hour ASPECTS and HB/RDW with the THRIVE score using the MFP algorithm to establish individualized predictions for IHM. Incorporating these variables improves predictive precision and accuracy for IHM. This novel prediction model aids in prioritizing care, influencing physician decision-making for treatment planning, and offering precise IHM risk information to the patient’s family. The variables used for calculation in the model are accessible, easily interpretable, cost-effective, and significantly enhance IHM prediction. Given the increasing prevalence of AIS patients receiving thrombolysis and limited stroke unit capacity, this model is crucial for accurate mortality risk assessment and effective patient stratification for intensive care. Additionally, transferring low-risk AIS patients to general medicine wards or nearby community hospitals can reduce congestion in stroke units and contribute to lower IHM rates after thrombolysis.

The THRIVE score is a well-established and validated tool for predicting stroke outcomes, particularly unfavorable outcomes and mortality in AIS patients with large vessel occlusion. ROC analysis revealed THRIVE score’s superior predictive performance over HIAT, HIAT-2, and SPAN-100 for 3-month mortality prediction, with an AuROC of 0.721 [21]. THRIVE-c calculation aimed to estimate adverse outcomes in Western patients receiving intravenous rt-PA. It surpassed the traditional THRIVE score, which categorizes individuals based on age and NIHSS scores into three groups. Using direct model estimation to assess outcome probability offers distinct advantages over the traditional THRIVE score. It achieves enhanced predictive ability by using continuous predictors instead of discretizing them for a simplified scoring system [9]. AuROC of the THRIVE score for predicting mortality ranges from approximately 0.7 to 0.8 across various studies [9, 22, 23], demonstrating a fair level of discrimination. These findings are consistent with the study conducted by Bo Shen et al. [24], which compared the prognostic performance of the THRIVE score, iScore score, and ASTRAL score in predicting poor outcomes in Chinese patients. The AuROC values for predicting 1-year mortality were 0.775, 0.830, and 0.809, respectively (all *p*<0.001). Contrary to our study findings, which revealed an AuROC value of 0.876 for the THRIVE-c model, previous studies have reported lower AuROC values (0.785) [9]. This disparity can be attributed to several factors, including different baseline patient characteristics, patients’ functional status, stroke severity on admission, and treatment approaches.

In routine clinical practice, the utilization of 24-hour ASPECTS and HB/RDW in predicting IHM among AIS patients treated with rt-PA remains limited due to the restricted availability of research data. However, there are supportive studies highlighting the impact of these predictors on stroke outcomes. For instance, Kong WY et al. [11] showed that 24-hour ASPECTS on NCCT was more accurate than baseline ASPECTS and changes in ASPECTS in predicting unfavorable functional outcomes after 3 months. (AuROC = 0.780). The SWIFT study demonstrated that utilizing 24-hour ASPECTS as a predictor for the therapeutic effects of reperfusion was the strongest indicator of clinical outcomes after 3 months of endovascular therapy [12].

Previous studies derived that the size of the infarct after an ischemic stroke may be related to the brain-heart axis, potentially causing cardiac dysfunction. ASPECTS is a mortality predictor, inversely correlating with stroke severity and infarct size. The strongest correlation is seen between infarct size (ASPECTS) and serum cardiac enzyme levels. A meta-analysis shows elevated cardiac biomarkers after acute ischemic stroke and intracerebral hemorrhage, indicating stroke-induced cardiac impairment and dysfunction [25]. Patients with extensive early ischemic changes may have limited benefits from thrombolysis or thrombectomy procedures. Moreover, these individuals may be more susceptible to treatment-related complications, such as intracerebral hemorrhage and malignant cerebral edema [26]. Brain edema worsens tissue dysfunction and is linked to increased morbidity and mortality [27].

The clinical significance of HB/RDW as a prognostic indicator for mortality is widely acknowledged, encompassing conditions like cardiovascular hospitalizations [16] and cancer [17] survival rates. Additionally, a recently conducted study highlighted the substantial association between HB/RDW and mortality in AIS patients diagnosed with AF. Particularly, there was an inverse relationship between overall mortality and HB/RDW values when the HB/RDW value was ≤9.74 [18]. On the other hand, as another parameter of HB/RDW, higher RDW, a measure of red blood cell size variation, independently predicts adverse outcomes in stroke patients. RDW levels exceeding 14% in AIS were associated with increased mortality within the first year [28]. We hypothesized a pathophysiological connection involving inflammation biomarkers in response to brain tissue necrosis. Elevated RDW levels inversely impact blood oxygen saturation and increase oxidative stress, potentially causing more severe and more progressive stroke by compromising brain oxygen supply [28].

Dichotomizing continuous variables in clinical prediction research leads to a significant loss of information, thereby diminishing the statistical power to identify any association between the variable and outcome. Furthermore, dichotomizing increases the likelihood of false positive outcomes, especially in cases with limited sample sizes, and masks any potential non-linear connection between the variable and the outcome [29]. In this study, we avoided dichotomizing continuous variables by using the MFP approach to construct the MVLR model. The key benefit of this approach is that it accounts for nonlinearity between the continuous variables and the logit transformation function, which can be present in certain cases. This leads to improved accuracy in the prediction model [30].

The mortality rate among thrombolysis patients in our study was higher at 18.8% compared to rates reported in previous trials (ranging from 9% to 15.7%) [25, 31]. The prediction of mortality is of great importance. Neuroimaging and laboratory data played critical roles in achieving accurate patient prognosis; however, they were not incorporated in the THRIVE-c score. The AuROC of 0.876 in our study indicates that the THRIVE-c calculation should be improved by incorporating 24-hour ASPECTS and HB/RDW. The novel predictive model demonstrated a substantial increase in AuROC to 0.980, which is considered an excellent level of discrimination.

### 4.1. Strength and limitations

The strengths of our study are as follows. Firstly, it is the first study to investigate the additional prognostic value of combining THRIVE score with 24-hour ASPECTS and HB/RDW for predicting IHM. Secondly, we proposed models using the MFP procedure and MVLR analysis, which resulted in more precise predictions. Thirdly, our study employed a population-based research design derived from real-world clinical practice. Fourth, both the 24-hour ASPECTS on NCCT and HB/RDW demonstrated significant predictive value, and our model is simple, cost-effective, and suitable for hospitals lacking advanced neuroimaging resources.

Our study had limitations. It was a single-center retrospective study, which limited its representativeness, and we excluded patients with incomplete data, potentially introducing selection bias. Further research should aim to develop a clinical model through a larger, prospective multicenter external validation study for clinical application. Second, our study focused exclusively on IHM without considering long-term outcomes. This limited scope may have underestimated overall mortality. However, clinical prediction rules in this context mostly prioritize IHM for stratifying AIS patient risk, which is crucial for planning early neurological monitoring and resource allocation in our hospital setting. Third, this study primarily focuses on AACIS patients receiving IV rt-PA due to limited EVT availability at our center, potentially impacting generalizability. Future research should explore clinical prediction scores and external validation for AIS patients undergoing other treatment strategies, like EVT. Fourth, the derived model relies on data from a single stroke center, limiting its representativeness. Further research should develop a clinical model through a larger, multicenter external validation study for clinical application. Fifth, and lastly, the study didn’t include all AIS patients, as it excluded those with posterior circulation ischemic stroke due to differences in ASPECTS grading, particularly in neuroanatomical location assessment. This limitation impacted the generalizability of the findings. Therefore, this model’s prediction isn’t suitable for decision-making in this subgroup.

## 5. Conclusions

In summary, the addition of 24-hour ASPECTS on NCCT and HB/RDW to the THRIVE score significantly enhances its predictive performance in determining IHM among AIS patients treated with rt-PA. Our prediction model provides more precise IHM prediction and can be applied in healthcare facilities with limited resources. This model will aid in improving treatment decision-making, stroke care planning, and the allocation of healthcare resources.

## Supporting information

S1 Table Comparative analysis of predictive values for IHM prediction in THRIVE-c and combined THRIVE-MFP models across different risk thresholds. (DOCX)

S2 Table Risk categorization and prognostic accuracy of the combined THRIVE-MFP model for predicting in-hospital mortality in thrombolyzed AACIS patients. (DOCX)

S3 Table Evaluation of incorporating 24-hour ASPECTS and HB/RDW with the THRIVE score using fractional polynomial transformation to predict 3-month mortality. (DOCX)

S4 Table Evaluation of incorporating 24-hour ASPECTS and HB/RDW with the THRIVE score using fractional polynomial transformation to predict 3-month poor functional outcome. (DOCX)

S1 Fig (a) Comparison of ROC curves and the evaluation of performance between the combined THRIVE-MFP model and THRIVE-c model for predicting 3-month mortality; (b) Model calibration plot illustrating the agreement between predicted odds and observed proportion of thrombolyzed AACIS patients.
(TIF)

S2 Fig (a) Comparison of ROC curves and the evaluation of performance between the combined THRIVE-MFP model and THRIVE-c model for predicting 3-month poor functional outcome; (b) Model calibration plot illustrating the agreement between predicted odds and observed proportion of thrombolyzed AACIS patients.
(TIF)

S3 Fig Calibration plot comparing the predicted risk of 3-month mortality from (a) THRIVE-c model, (b) combined THRIVE-MFP model, and (c) Internal validation of combined THRIVE-MFP model was performed using 500 bootstrap resamples. Note: The 45-degree straight line provides an ideal agreement between the observed and predicted probability. The vertical bars reflect the 95% CI of the actual probability.
(TIF)

S4 Fig Calibration plot comparing the predicted risk of 3-month poor functional outcome from (a) THRIVE-c model, (b) combined THRIVE-MFP model, and (c) Internal validation of combined THRIVE-MFP model was performed using 500 bootstrap resamples. Note: The 45-degree straight line provides an ideal agreement between the observed and predicted probability. The vertical bars reflect the 95% CI of the actual probability.
(TIF)

S5 Fig Decision curve analysis illustrating the net benefit of the combined THRIVE-MFP compared to the THRIVE-c model for predicting (a) 3-month mortality and (b) 3-month poor functional outcome.
(TIF)
