# Supplementary material for: Prognostic value of combining 24-hour ASPECTS and hemoglobin to red cell distribution width ratio to the THRIVE score in predicting in-hospital mortality among ischemic stroke patients treated with intravenous thrombolysis
Source: PLoS One. 2024 Jun 25;19(6):e0304765. doi: 10.1371/journal.pone.0304765 (PMC11198787; doi:10.1371/journal.pone.0304765)
Supplement: S1 Table — (DOCX) [file pone.0304765.s001.docx]

**Supporting information**

**S1 Table.** Comparative analysis of predictive values for IHM prediction in THRIVE-c and combined THRIVE-MFP models across different risk thresholds.

| **Predicted risk** | **IHM** | **Survived** | **Sensitivity (%)** | **Specificity (%)** | **PPV (%)** | **NPV (%)** | **LR+** | **Accuracy** |
| --- | --- | --- | --- | --- | --- | --- | --- | --- |
|  | **(n=65)** | **(n= 280)** | **(95%CI)** | **(95%CI)** | **(95%CI)** | **(95%CI)** | **(95%CI)** | **(95%CI)** |
| **Model A: THRIVE-c** **model** | | | | | | | | |
| ≥10% | 60 | 90 | 92.3 | 67.9 | 40 | 97.4 | 2.87 | 72.5 |
|  | 5 | 190 | (83 - 97.5) | (62 - 73.3) | (32.1 - 48.3) | (94.1 - 99.2) | (2.39 - 3.45) | (67.4 - 77.1) |
| ≥20% | 54 | 60 | 83.1 | 78.6 | 47.4 | 95.2 | 3.88 | 79.4 |
|  | 11 | 220 | (71.7 - 91.2) | (73.3 - 83.2) | (37.9 - 56.9) | (91.6 - 97.6) | (3.02 - 4.98) | (74.8 - 83.6) |
| ≥30% | 49 | 42 | 75.4 | 85.0 | 53.8 | 93.7 | 5.03 | 83.2 |
|  | 16 | 238 | (63.1 - 85.2) | (80.3 - 89) | (43.1 - 64.4) | (90 - 96.4) | (3.68 - 6.86) | (78.8 - 87) |
| ≥40% | 36 | 31 | 55.4 | 88.9 | 53.7 | 89.6 | 5.00 | 82.6 |
|  | 29 | 249 | (42.5 - 67.7) | (84.7 - 92.4) | (41.1 - 66) | (85.4 - 92.9) | (3.36 - 7.44) | (78.2 - 86.5) |
| ≥50% | 28 | 15 | 43.1 | 94.6 | 65.1 | 87.7 | 8.04 | 84.9 |
|  | 37 | 265 | (30.8 - 56) | (91.3 - 97) | (49.1 - 79) | (83.5 - 91.2) | (4.57 - 14.2) | (80.7 - 88.5) |
| **Model B: Combined THRIVE-MFP model** | | | | | | | | |
| ≥10% | 65 | 32 | 100.0 | 88.6 | 67.0 | 100 | 8.75 | 90.7 |
|  | 0 | 248 | (94.5 - 100) | (84.3 - 92.1) | (56.7 - 76.2) | (98.5 - 100) | (6.32 - 12.1) | (87.2 - 93.6) |
| ≥20% | 63 | 27 | 96.9 | 90.4 | 70.0 | 99.2 | 10.1 | 91.6 |
|  | 2 | 253 | (89.3 - 99.6) | (86.3 - 93.5) | (59.4 - 79.2) | (97.2 - 99.9) | (7 - 14.4) | (88.2 - 94.3) |
| ≥30% | 62 | 20 | 95.4 | 92.9 | 75.6 | 98.9 | 13.4 | 93.3 |
|  | 3 | 260 | (87.1 - 99) | (89.2 - 95.6) | (64.9 - 84.4) | (96.7 - 99.8) | (8.72 - 20.4) | (90.2 - 95.7) |
| ≥40% | 56 | 16 | 86.2 | 94.3 | 77.8 | 96.7 | 15.1 | 92.8 |
|  | 9 | 264 | (75.3 - 93.5) | (90.9 - 96.7) | (66.4 - 86.7) | (93.8 - 98.5) | (9.28 - 24.5) | (89.5 - 95.3) |
| ≥50% | 56 | 14 | 86.2 | 95.0 | 80.0 | 96.7 | 17.2 | 93.3 |
|  | 9 | 266 | (75.3 - 93.5) | (91.8 - 97.2) | (68.7 - 88.6) | (93.9 - 98.5) | (10.2 - 29.0) | (90.2 - 95.7) |

**Abbreviations:** ASPECTS, the Alberta stroke program early CT score; CI, confidence interval; THRIVE-c model, Total Health Risks in Vascular Events‐calculation model; combined THRIVE- MFP model, combined Total Health Risks in Vascular Events‐ multivariable fractional polynomial model; hemoglobin to red cell distribution width ratio; PPV, positive predictive value; NPV, negative predictive value; LR+, likelihood ratio positive.
