# Supplementary material for: Prognostic value of combining 24-hour ASPECTS and hemoglobin to red cell distribution width ratio to the THRIVE score in predicting in-hospital mortality among ischemic stroke patients treated with intravenous thrombolysis
Source: PLoS One. 2024 Jun 25;19(6):e0304765. doi: 10.1371/journal.pone.0304765 (PMC11198787; doi:10.1371/journal.pone.0304765)
Supplement: S2 Table — (DOCX) [file pone.0304765.s002.docx]

**Supporting information**

**S2 Table.** Risk categorization and prognostic accuracy of the combined THRIVE-MFP model for predicting in-hospital mortality in thrombolyzed AACIS patients.

| Predicted probability | IHM (n=65) | | Survived (n=280) | | PPV% (95%CI) | | LR+ (95%CI) | | Interpretation |
| --- | --- | --- | --- | --- | --- | --- | --- | --- | --- |
|  | n | (%) | n | (%) |  |  |  |  |  |
| <5% | 0 | (0.0) | 242 | (86.4) | 18.9 | (14.9 - 23.4) | 1.00 | (1.00 - 1.01) | Low risk |
| 5-25% | 2 | (3.1) | 17 | (6.1) | 63.1 | (53.0 - 72.4) | 7.37 | (5.48 - 9.90) | Intermediate risk |
| ≥25% | 63 | (96.9) | 21 | (7.5) | 75.0 | (64.4 - 83.8) | 12.90 | (8.55 - 19.50) | High risk |

**Abbreviations:** AACIS, acute anterior circulation ischemic stroke; CI, confidence interval; combined THRIVE- MFP model, combined Totaled Health Risks in Vascular Events ‐ multivariable fractional polynomial model; IHM, in-hospital mortality; LR+, likelihood ratio positive; PPV, positive predictive value.
