# Supplementary material for: Prognostic value of combining 24-hour ASPECTS and hemoglobin to red cell distribution width ratio to the THRIVE score in predicting in-hospital mortality among ischemic stroke patients treated with intravenous thrombolysis
Source: PLoS One. 2024 Jun 25;19(6):e0304765. doi: 10.1371/journal.pone.0304765 (PMC11198787; doi:10.1371/journal.pone.0304765)
Supplement: S3 Table — (DOCX) [file pone.0304765.s003.docx]

**Supporting information**

**S3 Table.** Evaluation of incorporating 24-hour ASPECTS and HB/RDW with the THRIVE score using fractional polynomial transformation to predict 3-month mortality.

| Model / Predictors Included | Optimal FP transformations | β | (95%CI) | *p*-value | AuROC (95%CI) | |
| --- | --- | --- | --- | --- | --- | --- |
| **Model A**^†^**: THRIVE-c** |  |  |  |  | 0.892 | (0.855-0.929) |
| Age | — | 0.025 | (0.003, 0.048) | 0.029 |  |  |
| NIHSS | — | 0.315 | (0.240, 0.390) | <0.001 |  |  |
| Diabetes mellitus | — | -0.134 | (-0.905, 0.637) | 0.733 |  |  |
| Hypertension | — | 0.249 | (-0.510, 1.009) | 0.520 |  |  |
| Atrial fibrillation | — | 0.539 | (-0.128, 1.206) | 0.113 |  |  |
| Intercept (constant) |  | -7.737 | (-9.677, -5.797) |  |  |  |
| **Model B**^‡^**: Combined THRIVE-MFP model** | |  |  |  | 0.947 | (0.921-0.974) |
| Age | Age - 61.777 | 0.037 | (0.008, 0.065) | 0.012 |  |  |
| NIHSS | NIHSS - 12.507 | 0.203 | (0.111, 0.296) | <0.001 |  |  |
| 24-hour ASPECTS | 24-hour ASPECTS-6.971 | -0.555 | (-0.720, -0.390) | <0.001 |  |  |
| HB/RDW | HB/RDW - 0.871 | -3.261 | (-5.418, -1.103) | <0.001 |  |  |
| Diabetes mellitus | Original binary form | -0.412 | (-1.355, 0.531) | 0.392 |  |  |
| Hypertension | Original binary form | 0.116 | (-0.766, 0.998) | 0.796 |  |  |
| Atrial fibrillation | Original binary form | -0.013 | (-0.837, 0.811) | 0.976 |  |  |
| Intercept (constant) |  | -2.446 | (-3.333, -1.559) |  |  |  |
| *p*-value* for difference of AuROC (Model A vs. Model B) | <0.001 | | | | | |

**Abbreviations:** AuROC, area under the receiver operating characteristic curve; ASPECTS, Alberta stroke program early CT score; FP, fractional polynomial; HB/RDW, hemoglobin to red cell distribution width ratio; IHM, in-hospital mortality; MFP, multivariable fractional polynomial; NIHSS, National Institute of Health Stroke Scale; THRIVE, Totaled Health Risks in Vascular Events.

The probability of 3-month mortality can be estimated using the combined THRIVE-MFP model through the following equation: e^z^/(1 + e^z^), where z = -2.446+ 0.037 (Age - 61.777) + 0.203 (NIHSS - 12.507) + -0.555 (24-hour ASPECTS-6.971) + -3.261 (HB/RDW - 0.871) + -0.412 (DM: No=0 or Yes=1) + 0.116 (HTN: No=0 or Yes=1) + -0.013 (AF: No=0 or Yes=1)

^†^Model A includes THRIVE-c model; ^‡^Model B includes THRIVE score with 24-hour ASPECTS and HB/RDW using MFP algorithm (combined THRIVE- MFP model).

**p*-value for significant difference in AuROC using method proposed by DeLong et al.

Hosmer-Lemeshow test was used (*p*-value= 0.407 for model A and *p*-value= 0.703 for model B).
