# Supplementary material for: Prognostic value of combining 24-hour ASPECTS and hemoglobin to red cell distribution width ratio to the THRIVE score in predicting in-hospital mortality among ischemic stroke patients treated with intravenous thrombolysis
Source: PLoS One. 2024 Jun 25;19(6):e0304765. doi: 10.1371/journal.pone.0304765 (PMC11198787; doi:10.1371/journal.pone.0304765)
Supplement: S4 Table — (DOCX) [file pone.0304765.s004.docx]

**Supporting information**

**S4 Table.** Evaluation of incorporating 24-hour ASPECTS and HB/RDW with the THRIVE score using fractional polynomial transformation to predict 3-month poor functional outcome.

| Model / Predictors Included | Optimal FP transformations | β | (95%CI) | *p*-value | AuROC (95%CI) | |
| --- | --- | --- | --- | --- | --- | --- |
| **Model A**^†^**: THRIVE-c** |  |  |  |  | 0.853 | (0.813, 0.894) |
| Age | — | 0.035 | (0.016, 0.055) | <0.001 |  |  |
| NIHSS | — | 0.253 | (0.195, 0.311) | <0.001 |  |  |
| Diabetes mellitus | — | 0.069 | (-0.561, 0.699) | 0.830 |  |  |
| Hypertension | — | 0.251 | (-0.375, 0.878) | 0.432 |  |  |
| Atrial fibrillation | — | 0.271 | (-0.328, 0.870) | 0.375 |  |  |
| Intercept (constant) |  | -6.123 | (-7.655, -4.592) |  |  |  |
| **Model B**^‡^**: Combined THRIVE-MFP model** | |  |  |  | 0.910 | (0.880, 0.940) |
| Age | Age-61.777 | 0.042 | (0.019, 0.064) |  |  |  |
| NIHSS | NIHSS-12.507 | 0.129 | (0.059, 0.198) |  |  |  |
| 24-hour ASPECTS | 24-hour ASPECTS-6.971 | -0.523 | (-0.681, -0.366) |  |  |  |
| HB/RDW | HB/RDW - 0.871 | -1.581 | (-3.220, 0.058) |  |  |  |
| Diabetes mellitus | Original binary form | -0.016 | (-0.732, 0.699) |  |  |  |
| Hypertension | Original binary form | 0.306 | (-0.400, 1.012) |  |  |  |
| Atrial fibrillation | Original binary form | -0.093 | (-0.792, 0.606) |  |  |  |
| Intercept (constant) |  | -0.624 | (-1.240, -0.007) |  |  |  |
| *p*-value* for difference of AuROC (Model A vs. Model B) | <0.001 | | | | | |

**Abbreviations:** AuROC, area under the receiver operating characteristic curve; ASPECTS, Alberta stroke program early CT score; FP, fractional polynomial; HB/RDW, hemoglobin to red cell distribution width ratio; IHM, in-hospital mortality; MFP, multivariable fractional polynomial; NIHSS, National Institute of Health Stroke Scale; THRIVE, Totaled Health Risks in Vascular Events.

The probability of 3-month poor functional outcome can be estimated using the combined THRIVE-MFP model through the following equation: e^z^/(1 + e^z^), where z = -0.624 + 0.042 (Age - 61.777) + 0.129 (NIHSS - 12.507) + -0.523 (24-hour ASPECTS-6.971) + -1.581 (HB/RDW - 0.871) + -0.016 (DM: No=0 or Yes=1) + 0.306 (HTN: No=0 or Yes=1) + -0.093 (AF: No=0 or Yes=1)

^†^Model A includes THRIVE-c model; ^‡^Model B includes THRIVE score with 24-hour ASPECTS and HB/RDW using MFP algorithm (combined THRIVE- MFP model).

**p*-value for significant difference in AuROC using method proposed by DeLong et al.

Hosmer-Lemeshow test was used (*p*-value= 0.445 for model A and *p*-value= 0.209 for model B).
