# Supplementary figures and images for: Prognostic value of combining 24-hour ASPECTS and hemoglobin to red cell distribution width ratio to the THRIVE score in predicting in-hospital mortality among ischemic stroke patients treated with intravenous thrombolysis
Source: PLoS One. 2024 Jun 25;19(6):e0304765. doi: 10.1371/journal.pone.0304765 (PMC11198787; doi:10.1371/journal.pone.0304765)

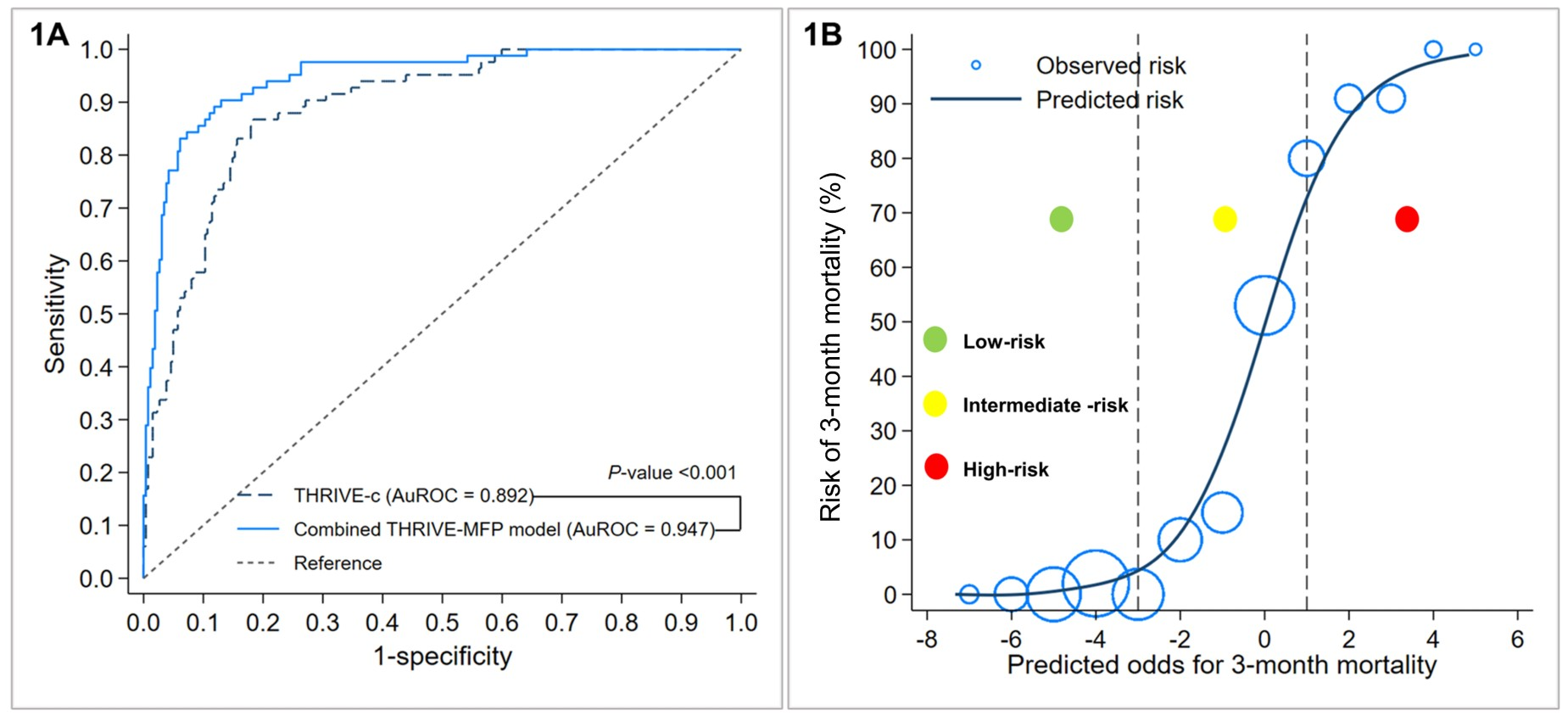

Supplement: S1 Fig — (a) Comparison of ROC curves and the evaluation of performance between the combined THRIVE-MFP model and THRIVE-c model for predicting 3-month mortality; (b) Model calibration plot illustrating the agreement between predicted odds and observed proportion of thrombolyzed AACIS patients. (TIF) [file pone.0304765.s005.tif]

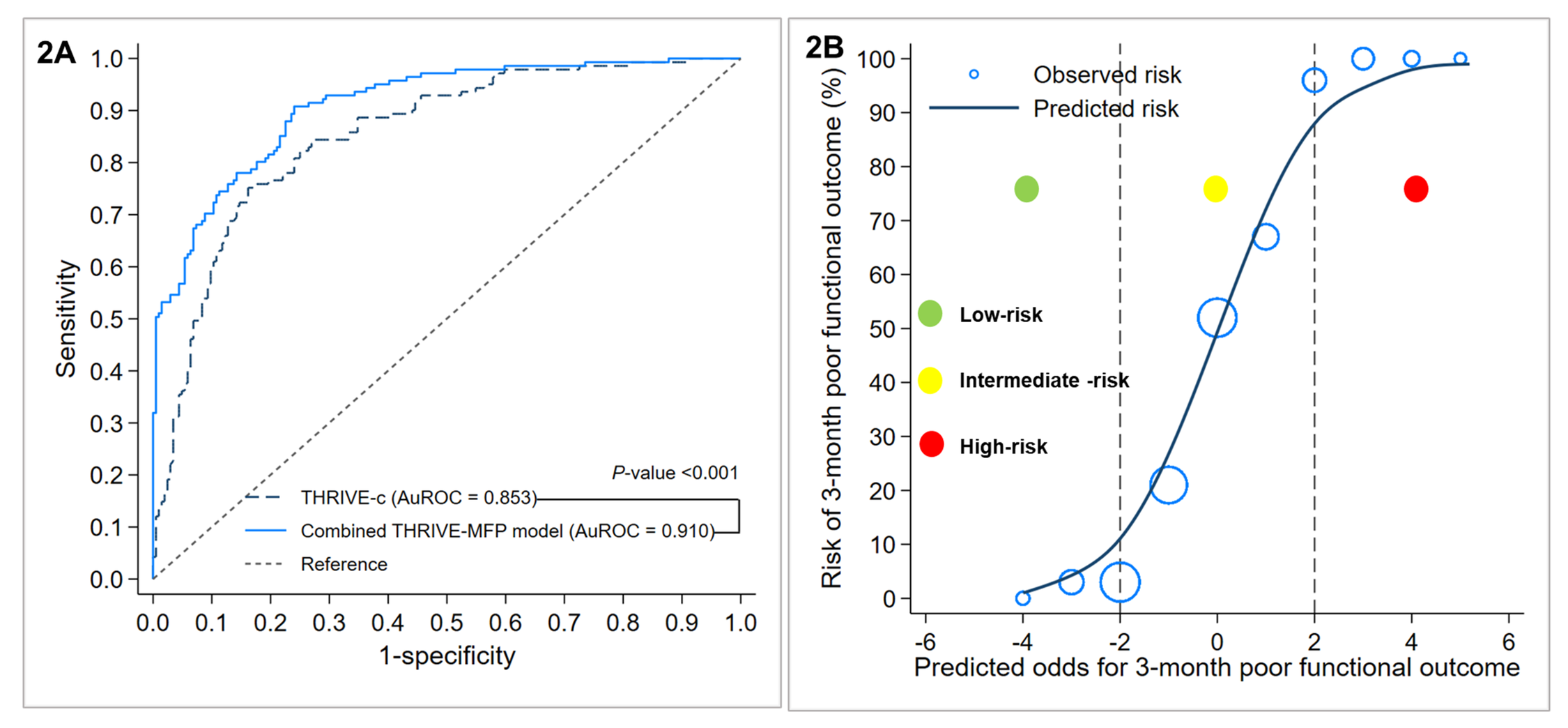

Supplement: S2 Fig — (a) Comparison of ROC curves and the evaluation of performance between the combined THRIVE-MFP model and THRIVE-c model for predicting 3-month poor functional outcome; (b) Model calibration plot illustrating the agreement between predicted odds and observed proportion of thrombolyzed AACIS patients. (TIF) [file pone.0304765.s006.tif]

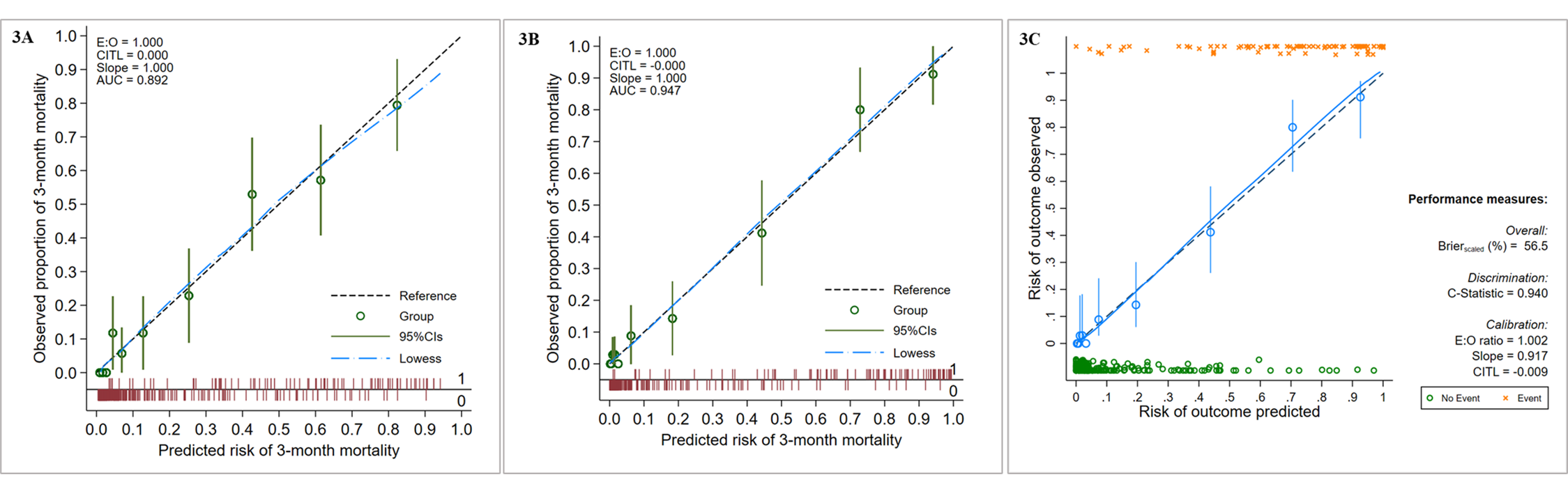

Supplement: S3 Fig — Calibration plot comparing the predicted risk of 3-month mortality from (a) THRIVE-c model, (b) combined THRIVE-MFP model, and (c) Internal validation of combined THRIVE-MFP model was performed using 500 bootstrap resamples. Note: The 45-degree straight line provides an ideal agreement between the observed and predicted probability. The vertical bars reflect the 95% CI of the actual probability. (TIF) [file pone.0304765.s007.tif]

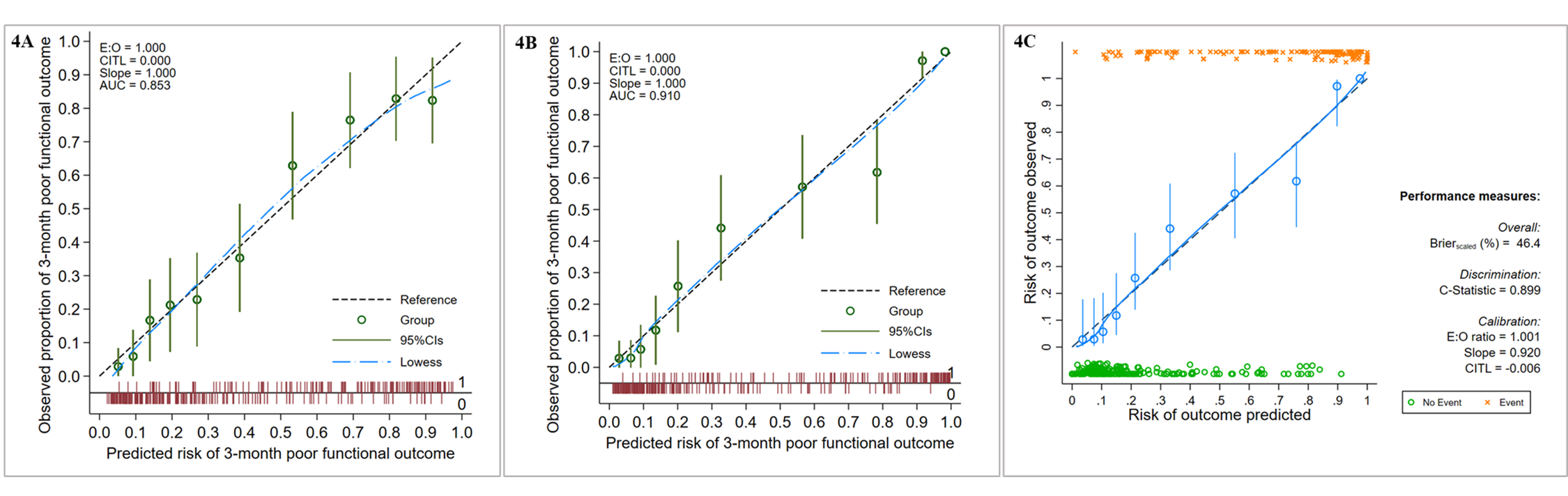

Supplement: S4 Fig — Calibration plot comparing the predicted risk of 3-month poor functional outcome from (a) THRIVE-c model, (b) combined THRIVE-MFP model, and (c) Internal validation of combined THRIVE-MFP model was performed using 500 bootstrap resamples. Note: The 45-degree straight line provides an ideal agreement between the observed and predicted probability. The vertical bars reflect the 95% CI of the actual probability. (TIF) [file pone.0304765.s008.tif]

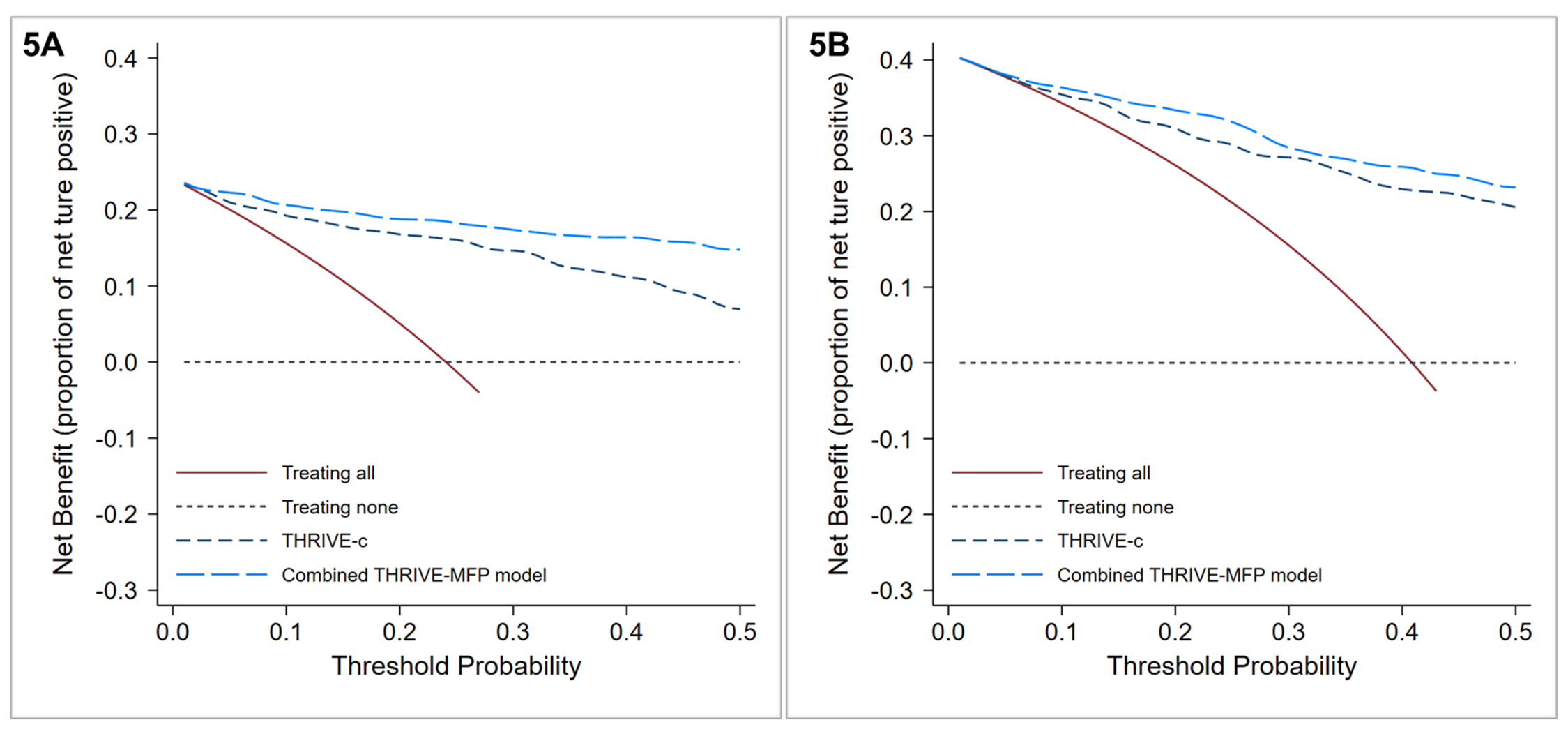

Supplement: S5 Fig — Decision curve analysis illustrating the net benefit of the combined THRIVE-MFP compared to the THRIVE-c model for predicting (a) 3-month mortality and (b) 3-month poor functional outcome. (TIF) [file pone.0304765.s009.tif]
